# Deciphering intrafamilial phenotypic variability by exome sequencing in a Bardet–Biedl family

**DOI:** 10.1002/mgg3.50

**Published:** 2013-12-03

**Authors:** María González-del Pozo, Cristina Méndez-Vidal, Javier Santoyo-Lopez, Alicia Vela-Boza, Nereida Bravo-Gil, Antonio Rueda, Luz García-Alonso, Carmen Vázquez-Marouschek, Joaquín Dopazo, Salud Borrego, Guillermo Antiñolo

**Affiliations:** 1 Department of Genetics, Reproduction and Fetal Medicine, Institute of Biomedicine of Seville, University Hospital Virgen del Rocio/Consejo Superior de Investigaciones Científicas/University of SevilleSeville, Spain; 2 Centre for Biomedical Network Research on Rare Diseases (CIBERER)Seville, Spain; 3 Medical Genome Project, Genomics and Bioinformatics Platform of Andalusia (GBPA)Seville, Spain; 4 Department of Computational Genomics, Centro de Investigación Príncipe Felipe (CIPF)Valencia, Spain; 5 Department of Ophthalmology, University Hospital Virgen del RocíoSeville, Spain; 6 Functional Genomics Node (INB), CIPFValencia, Spain

**Keywords:** Bardet–Biedl Syndrome, intrafamilial variability, *MKKS*, NGS, *NPHP4*

## Abstract

Bardet–Biedl syndrome (BBS) is a model ciliopathy characterized by a wide range of clinical variability. The heterogeneity of this condition is reflected in the number of underlying gene defects and the epistatic interactions between the proteins encoded. BBS is generally inherited in an autosomal recessive trait. However, in some families, mutations across different loci interact to modulate the expressivity of the phenotype. In order to investigate the magnitude of epistasis in one BBS family with remarkable intrafamilial phenotypic variability, we designed an exome sequencing–based approach using SOLID 5500xl platform. This strategy allowed the reliable detection of the primary causal mutations in our family consisting of two novel compound heterozygous mutations in McKusick–Kaufman syndrome (*MKKS*) gene (p.D90G and p.V396F). Additionally, exome sequencing enabled the detection of one novel heterozygous *NPHP*4 variant which is predicted to activate a cryptic acceptor splice site and is only present in the most severely affected patient. Here, we provide an exome sequencing analysis of a BBS family and show the potential utility of this tool, in combination with network analysis, to detect disease-causing mutations and second-site modifiers. Our data demonstrate how next-generation sequencing (NGS) can facilitate the dissection of epistatic phenomena, and shed light on the genetic basis of phenotypic variability.

## Introduction

Ciliopathies are an emerging group of clinical disorders characterized by large genetic heterogeneity and clinical overlap. Due to the ubiquitous nature of the primary cilium, ciliopathies can affect many organ systems (Fliegauf et al. 2007). Bardet–Biedl syndrome (BBS; MIM 209900) is considered a model of ciliopathy (Badano et al. 2006a), and its prevalence varies from 1:160,000 in Europe (Klein and Ammann 1969; Beales et al. 1997) to 1:13,500 and 1:17,500 in Kuwait and Newfoundland, respectively (Farag and Teebi 1989; Green et al. 1989). BBS is a pleiotropic disorder that has variable expressivity and a wide range of clinical variability observed both within and between families (Beales et al. 1999). Primary features include retinitis pigmentosa (RP), polydactyly, obesity, genital defects, renal anomalies, and learning disabilities. Secondary features include speech delay, developmental delay, diabetes mellitus, dental anomalies, congenital heart disease, brachydactyly/syndactyly, ataxia/poor coordination, and anosmia/hiposmia (Forsythe and Beales 2013).

The clinical diagnosis of BBS requires the presence of at least four primary or three primary and two secondary features (Beales et al. 1999; Rooryck and Lacombe 2008; Putoux et al. 2012). The spectrum of clinical features of BBS shares common characteristics with other ciliopathies such as Joubert syndrome (JBTS; MIM 213300), Leber congenital amaurosis (LCA; MIM 204000), McKusick–Kaufman syndrome (MKKS; MIM 236700), Meckel–Gruber syndrome (MKS; MIM 249000), nephronophthisis (NPH; MIM 256100), orofaciodigital syndrome type 1 (OFD1; MIM 311200), and Senior–Løken syndrome (SLS; MIM 266900). Although these are considered as distinct clinical entities, in many cases it is very difficult to assign a specific clinical diagnosis due to phenotypic overlap. Accordingly, to the phenotypic overlap, genetic overlap also exists. Thus, mutations in *MKKS* (MIM 604896) also cause MKKS – characterized by genitourinary malformations (hydrometrocolpos), polydactyly, and more rarely gastrointestinal abnormalities (McKusick et al. 1964; Stone et al. 2000).

To date, mutations in 18 genes have been associated with BBS (Retinal Information Network website. Available: https://sph.uth.edu/retnet/) accounting for ∼70% of affected individuals (Muller et al. 2010). BBS is typically inherited in an autosomal recessive manner. However, the involvement of mutations in more than one locus has been substantially growing. Digenic triallelic inheritance has been reported in some patients with three mutations across two BBS loci that interact to cause disease (Katsanis et al. 2001; Badano et al. 2003; Beales et al. 2003). In addition, the presence of second-site modifiers that may modulate the expression of the clinical phenotype has been proposed to explain, in part, the significant inter-and intrafamilial clinical variability in BBS (Katsanis 2004; Badano et al. 2006b; Khanna et al. 2009).

In the last few years, the introduction of the next-generation sequencing (NGS) has revolutionized clinical genetics, making whole-exome sequencing (WES) a rapid way to elucidate the genetic basis of clinically and genetically heterogeneous Mendelian disorders (Bamshad et al. 2011; Ionita-Laza et al. 2011; Rabbani et al. 2012). At present, it is expected that NGS, in combination with network analysis (Minguez et al. 2009) and other advanced bioinformatics tools that allows prioritizing candidate genes because of their functional relationships (Ideker and Sharan 2008), will play an increasingly important role in the diagnosis of complex and oligogenic disorders. Moreover, WES provides a unique possibility for investigating the presence of additional mutations that may modify the expressivity of BBS phenotype. Other authors have been interested in determining the contribution of epistasis in BBS (Katsanis et al. 2002; Hichri et al. 2005; Smaoui et al. 2006; Abu-Safieh et al. 2012), but their approaches have been based on Sanger sequencing of known BBS loci (Katsanis et al. 2002; Hichri et al. 2005; Smaoui et al. 2006; Abu-Safieh et al. 2012) or on targeted exon capture sequencing of a number of genes (Redin et al. 2012).

In this study, we conducted a WES approach in a Spanish family with significant intrafamilial variability in the BBS phenotype, from full blown to relatively mild forms of BBS. Using this technology, compound heterozygous mutations have been found in *MKKS* and the contribution of additional changes has been dissected.

## Material and Methods

### Subjects and clinical assessment

**Figure 1:**
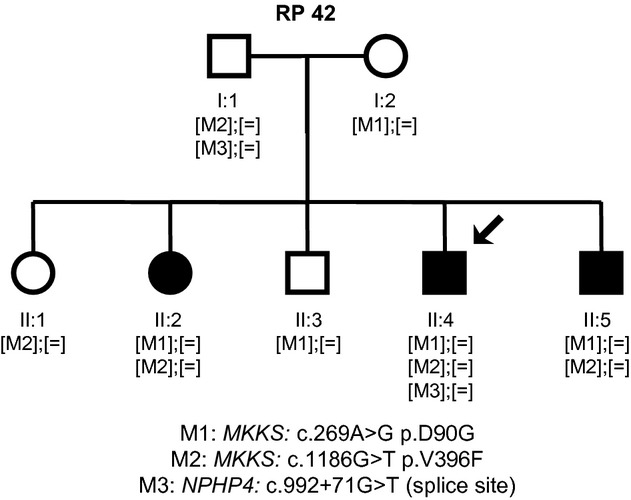
Family cosegregation analysis. RP42 family tree showing the segregation of the sequence variants identified during the molecular analysis of *MKKS* and *NPHP4*. [M];[M]: homozygous; [M];[=]: heterozygous.

Our study involved one Spanish family (RP42) comprising three affected and four unaffected individuals (Fig. 1), all derived from the Ophthalmology Department to our Genetic Department. The study was carried out in accordance with the tenets of the Declaration of Helsinki and the ethical guidelines of our institutions. A group of 200 matching control individuals was also recruited. Written informed consent was obtained from all participants.

Clinical diagnosis of retinal degeneration was based on visual acuity, fundus photography, computerized testing of central and peripheral visual fields, and electroretinography (ERG) findings. Typical ocular features include initial night blindness, restriction of visual field, bone spicule pigmentation, attenuation of retinal vessels, waxy disk pallor, and abnormal ERG findings in a rod-cone pattern when recordable.

All subjects underwent a peripheral blood extraction for genomic DNA isolation from leukocytes using standard protocols. DNA samples from individuals I:1, I:2, II:3, and II:4 were processed for NGS.

After the identification of the *MKKS* gene defects, affected individuals underwent clinical reevaluation focused on the identification of extraocular features associated with BBS.

### Description of DNA library preparation and sequencing

Library preparation and exome capture were performed according to a protocol based on the Baylor College of Medicine protocol version 2.1 with several modifications. Briefly, 5 *μ*g of input genomic DNA was sheared, end repaired, and ligated with specific adaptors. A fragment size distribution ranging from 160 bp to 180 bp after shearing and 200–250 bp after adaptor ligation was verified by Bioanalyzer (Agilent Technologies, Santa Clara, CA). The library was amplified by precapture linker-mediated polymerase chain reaction (LM-PCR) using FastStart High Fidelity PCR System (Roche, Indianapolis, IN). After purification, 2 *μ*g of LM-PCR product was hybridized to NimbleGen SeqCap EZ Exome libraries V3. After washing, amplification was performed by postcapture LM-PCR using FastStart High Fidelity PCR System (Roche). Capture enrichment was measured by qPCR according to NimbleGen protocol. The successfully captured DNA was measured by Quant-iT™ PicoGreen® dsDNA reagent (Invitrogen, Carlsbad, CA) and subjected to standard sample preparation procedures for sequencing with SOLiD 5500xl platform as recommended by the manufacturer. Shortly, emulsion PCR was performed on E80 scale (about 1 billion template beads) using a concentration of 0.616 pm of enriched captured DNA. After breaking and enrichment, about 276 million enriched template beads were sequenced per lane on a six-lane SOLiD 5500xl slide.

### Analysis of data from deep sequencing

SOLiD 5500xl reads were aligned against the human genome reference (hg19) using the program BFAST (Blat-like Fast Accurate Search Tool) allowing reads to map only to a unique position in the reference genome. Improperly mapped reads were filtered out with the SAMtools package, which was also used to sort SAM files and to generate and index BAM files. Variant calling was performed with the software GATK (Genome Analysis Toolkit) taking into account variants from NCBI database of Single Nucleotide Polymorphisms (dbSNP) for recalibration and realignment. Secondary analysis was performed by a custom shell script that queries VARIANT database (Medina et al. 2012), which includes SIFT (Kumar et al. 2009) and Polyphen-2 (Adzhubei et al. 2010) scores, to annotate all single-nucleotide variations (SNVs) and small insertions and deletions (INDELS). Variants with an allele frequency higher than 5% in the 1000 Genomes Project database were discarded. Only, exonic variants which produce a synonymous change in the open reading frame were discarded, whereas other type of variants in the exomes and variants in splice sites were kept for further analysis. Then, variants found in affected individuals were compared with variants present in not affected relatives. A last step was performed to compare the remaining variants with variants obtained from a group of healthy controls from the same local population as the family in study obtained from The Medical Genome Project (http://www.medicalgenomeproject.com). Finally, genes with variants in both alleles present in affected but not in healthy individuals of the family nor in the control local population and with a variant only in one allele in the carrier were ranked based on the analysis of the interactome, using NetworkMiner(Garcia-Alonso et al. 2012), to generate a list of candidate genes.

### Verification and assessment of the pathogenicity of variants

Each predicted disease-causing variant was confirmed by Sanger sequencing, and cosegregation analysis was performed in the rest of the family members DNA samples.

As mentioned above, we used Polyphen-2 and SIFT scores to evaluate the potential impact of novel missense substitutions on the function of the encoded protein. Evolutionary conservation across species was assessed through the alignment of orthologous MKKS protein sequences *(Pan troglodytes*,*Mus musculus*,*Canis lupus familiaris*,*Gallus gallus*,*Xenopus tropicalis*, and *Danio rerio*) with the human MKKS protein sequence, using Clustal Omega Tool (Sievers et al. 2011). Furthermore, splice site tool Prediction by Neural Network (Reese 1997; Reese et al. 1997), exonic splicing enhancer prediction programs ESE Finder (Cartegni et al. 2003; Smith et al. 2006), and NetGene2 server (Hebsgaard et al. 1996) were applied to estimate the pathogenic nature of intronic sequence variants that could affect the splicing process. The correct nomenclature for mutation was checked applying Mutalyzer (Wildeman et al. 2008) using the corresponding Genbank reference sequences (*MKKS*; NG_009109.1 and *NPHP4*; NG_011724.2). Novel variants included in this article were submitted to the respective Locus Specific Database (LSDB) (http://grenada.lumc.nl/LOVD2/eye/home.php?select_db=MKKS).

### Network analysis

Network enrichment analysis has been performed using the program SNOW (Minguez et al. 2009) included in the Babelomics (Medina et al. 2010) package (http://www.babelomics.org). Genes are mapped onto the interactome (obtained from the STRING [Franceschini et al. 2013] database), and the subnetwork connecting them is obtained. Several relevant parameters are calculated for this subnetwork, such as the connectivity or the number of components. An empirical distribution of the random expectation of these parameters is obtained by repeatedly sampling random sets of the same number of genes from the complete genome and calculating the average connectivity of their corresponding subnetworks. Thus, real values of the parameters obtained for the genes analyzed can be contrasted with respect to their random expectations (Minguez et al. 2009; Minguez and Dopazo 2010).

## Results

### Clinical assessment

The clinical findings of the affected individuals (II:2, II:4, and II:5) are reported in Table1. Ocular manifestations of the disease in the three siblings were fairly typical of an early onset and severe form of RP. Night blindness was reported from the first decade. Thereafter, the disease rapidly progressed, and by the age of 20, II:2, II:4, and II:5 were severely visually disabled. The fundus examination showed the typical signs of RP, including pale waxy disks, attenuation of the retinal vessels, and bone spicule pigmentation in the midperiphery. Photopic and scotopic ERGs were extinguished (II:2) or diminished (II:4) on examination at ages 21 and 16, respectively. Patient II:4 showed extraocular features commonly associated with BBS such as postaxial polydactyly, overweight, polycystic kidney, learning disabilities, and mild psychomotor delay. Patients II:2 and II:5 exhibited a less severe phenotype consisting of RP, postaxial polydactyly, and mild learning disabilities. Subject II:4 underwent renal transplantation due to polycystic kidney disease, whereas the other two did not manifest any renal abnormalities. Therefore, patient II:4 fulfilled the criteria for the diagnosis of classic BBS, whereas II:2 and II:5 were diagnosed of mild BBS.

**Table 1:** Summary of phenotype features documented in the affected members of the family RP 42.

Feature	II:2	II:4	II:5
First symptom	Night blindness	Night blindness	Night blindness
Onset age	3	10	8
Primary features			
Retinitis pigmentosa	+	+	+
Postaxial polydactyly	Both feet	Both feet	One foot
Weight gain anomaly	Normal weight	Obese	Overweight
Genital defects, hydrometrocolpos	−	−	−
Learning disabilities	+	+	+
Renal anomalies			
Kidney cysts	−	+	−
Kidney transplant	−	+	−
Secondary features			
Poor coordination	−	+	−
Developmental delay	−	−	−
Speech delay	−	−	−
Brachydactyly/Syndactyly	−	−	−
Dental anomalies			
Teeth crowding	−	−	−
Congenital heart disease	−	−	−

### Identification of causal mutations

**Figure 2:**
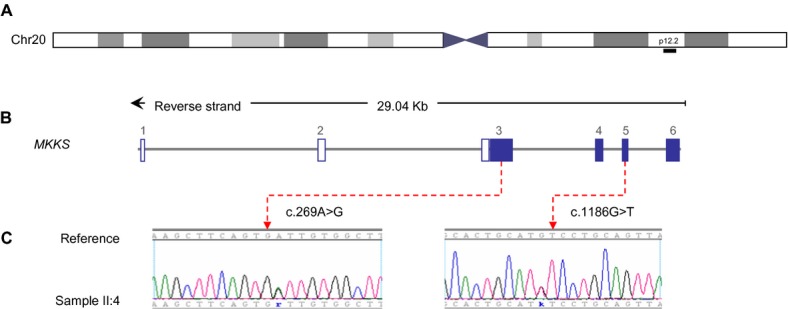
Detection of novel mutations in the *MKKS* gene. (A) Chromosome overview of the chromosome 20, *MKKS* is mapped on region 20p12.2 (black bar). (B) *MKKS* spans approximately 29 Kb and is composed of six exons. Filled boxes reflect coding exons (3–6) and unfilled boxes reflect UTR. (C) Electropherogram depiction of the index patient (II:4) confirming the heterozygous mutations in exons 3 and 5 of MKKS gene. IUPAC SNP codes used to designate heterozygous substitutions (“k” from Keto for G/T and “r” from puRine for A/G). *MKKS* Genbank accession number: NG_009109.1.

**Figure 3:**
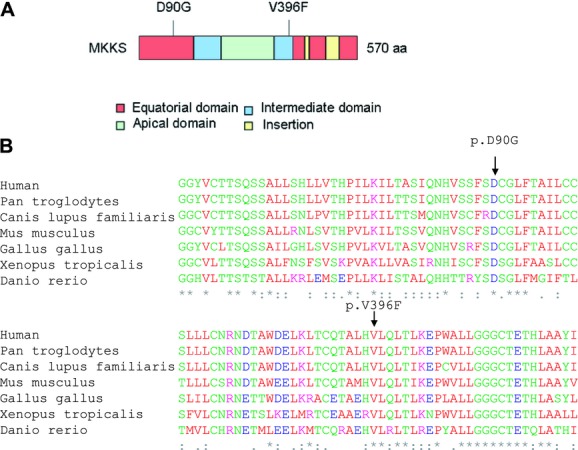
MKKS protein depiction. (A) Schematic representation of the identified variants within the MKKS domains, including the typical chaperonin group II domains (equatorial, intermediate, and apical). The domain organization was modified from (Stoetzel et al. 2007). (B) Alignment of the orthologs from different species showing conservation of the mutated residues. An * (asterisk) indicates positions which have a single, fully conserved residue. A: (colon) indicates conservation between groups of strongly similar properties. A. (period) indicates conservation between groups of weakly similar properties.

Upon exome sequencing, 43,570 sequence variants were identified in patient II:4 (Table2). The two most probably pathogenic mutations located on exons 3 and 5 of *MKKS* gene (Fig. 2) have never been reported in public variant databases such as dbSNP, Exome Variant Server (http://evs.gs.washington.edu/EVS/), or 1000 genomes (http://www.1000genomes.org/). Sanger sequencing of these exons confirmed the novel missense variants (c.269A>G; p.D90G and c.1186G>T; p.V396F) in a compound heterozygous state in the affected siblings. The variants cosegregated with the disease in the entire family (Fig. 1) and were absent in 400 control chromosomes. Mutation D90G lay within the predicted equatorial domain and mutation V396F lay within the predicted intermediate domain which connects the equatorial and the apical domains via flexible hinges (Stone et al. 2000) (Fig. 3A). Alignment of *MKKS* amino acid sequence of various orthologs showed that the substituted residues (aspartic acid at position 90 and valine at position 396) are highly conserved across species from different evolutionary branches (Fig. 3B). In silico tools predicted that both p.D90G and p.V396F are probably damaging by Polyphen-2 (score = 1 for p.D90G and score = 0.987 for p.V396F) and SIFT (score = 0.01 for p.D90G and score = 0 for p.V396F).

**Table 2:** Variants identified by exome sequencing in the RP 42 family.

	I:1	I:2	II:3	II:4
Total SNVs	45,068	42,243	43,269	43,570
Nonsynonymous SNVs	6114	5855	5892	5942
Filtered dbSNP	5979	5735	5756	5821
Filtered dbSNP and 1000 g	5913	5692	5710	5760
Filtered dbSNP and 1000 g and predicted deleterious	357	373	355	367

SNVs, single-nucleotide variations.

### Epistasis evaluation

Whole-exome data were subjected to exhaustive evaluation paying special attention in the identification of additional variants that may be acting as second-site modifiers making patient's II:4 BBS phenotype more severe. Although several SNPs were identified in the remaining 16 known BBS genes: *BBS1* (MIM 209901)*, BBS2* (MIM 606151)*, ARL6* (*BBS3*; MIM 608845), *BBS4* (MIM 600374), *BBS5* (MIM 603650), *MKKS* (*BBS6*), *BBS7* (MIM 607590), *TTC8* (*BBS8*; MIM 608132), *BBS9* (MIM 607968), *BBS10* (MIM 610148), *TRIM32* (*BBS11*; MIM 602290), *BBS12* (MIM 610683), *MKS1* (*BBS13*; MIM 609883), *CEP290* (*BBS14*; MIM 610142), *SDCCAG8* (*BBS16*; MIM 613524), *LZTFL1* (*BBS17*; MIM 606568), and *INPP5E* (MIM 613037) (Table S1); none of them was predicted to be pathogenic.

To explore the possibility that the polycystic kidney disease (PKD), affecting only patient II:4, may be caused by mutations in other loci such as PKD genes: *PKD1* (MIM 601313)*, PKD2* (MIM 173910), and *PKHD1* (MIM 606702); or nephronophthisis genes: *NPHP1* (MIM 607100), *INVS* (*NPHP2*; MIM 243305), *NPHP3* (MIM 608002)*, NPHP4* (MIM 607215), *IQCB1 (NPHP5*; MIM 609237), *GLIS2* (*NPHP7*; MIM 608539), *RPGRIP1L* (*NPHP8*; MIM 610937), *NEK8* (*NPHP9*; MIM 609799), *NEK1* (MIM 604588), *MKS1* (MIM 609883), and *TMEM67* (*NPHP11*; MIM 609884) (Table S1), we evaluated the exome data and found one novel variant in intron 8-9 of the *NPHP4* gene (c.992 + 71G>T). This variant has never been reported in databases such as dbSNP and EVS. As coverage information for this position was not clear in the EVS data set, we checked the absence of c.992 + 71G>T in additional genomic databases which also include intronic variants (1000 genomes and 5000 genomes). Family segregation showed the presence of the heterozygous *NPHP4* variant only in patient II:4 and in his unaffected parent (I:1) (Fig. 1). In silico tools predicted that the substitution of a G > T in this position activates a cryptic splice acceptor site in the intron 8-9 of *NPHP4* (Table3). The complete coding sequence was scanned to eliminate the possibility of the presence of other mutation in *NPHP4*. The absence in 400 control chromosomes and the results of the *in silico* prediction supported the pathogenic role of the *NPHP4* c.992 + 71G>T variant.

**Table 3:** Splice acceptor site prediction scores for *NPHP4* c.992 + 71G>T mutation versus wild type.

Allele	Wild type aggtcatttgt**g**catgtcaggtgt	c.992 + 71G>T intronEXON aggtcatttgttcatgtcagGTGT
NNSPLICE	0	0.71
ESEfinder		
3SS_U2_human (threshold: 6.632)	0	7.735500
3SS_U2_mouse (threshold: 6.724)	0	7.26420
BranchSite (threshold: 0)	0	2.07090 (tgttcat)
NetGene2	0	0.28

Sequence variants are highlighted in bold; potential splice acceptor site are underlined; predicted exonic sequence is in capital letters. 3SS_U2_Human: 3's splice sites (acceptor) of human (U2 type). 3SS_U2_mouse: 3's splice sites (acceptor) of mouse (U2 type). Branch site: mammalian branch site (U2 type). *NPHP4* Genbank accession number: NG_011724.2.

### Network analysis

**Figure 4:**
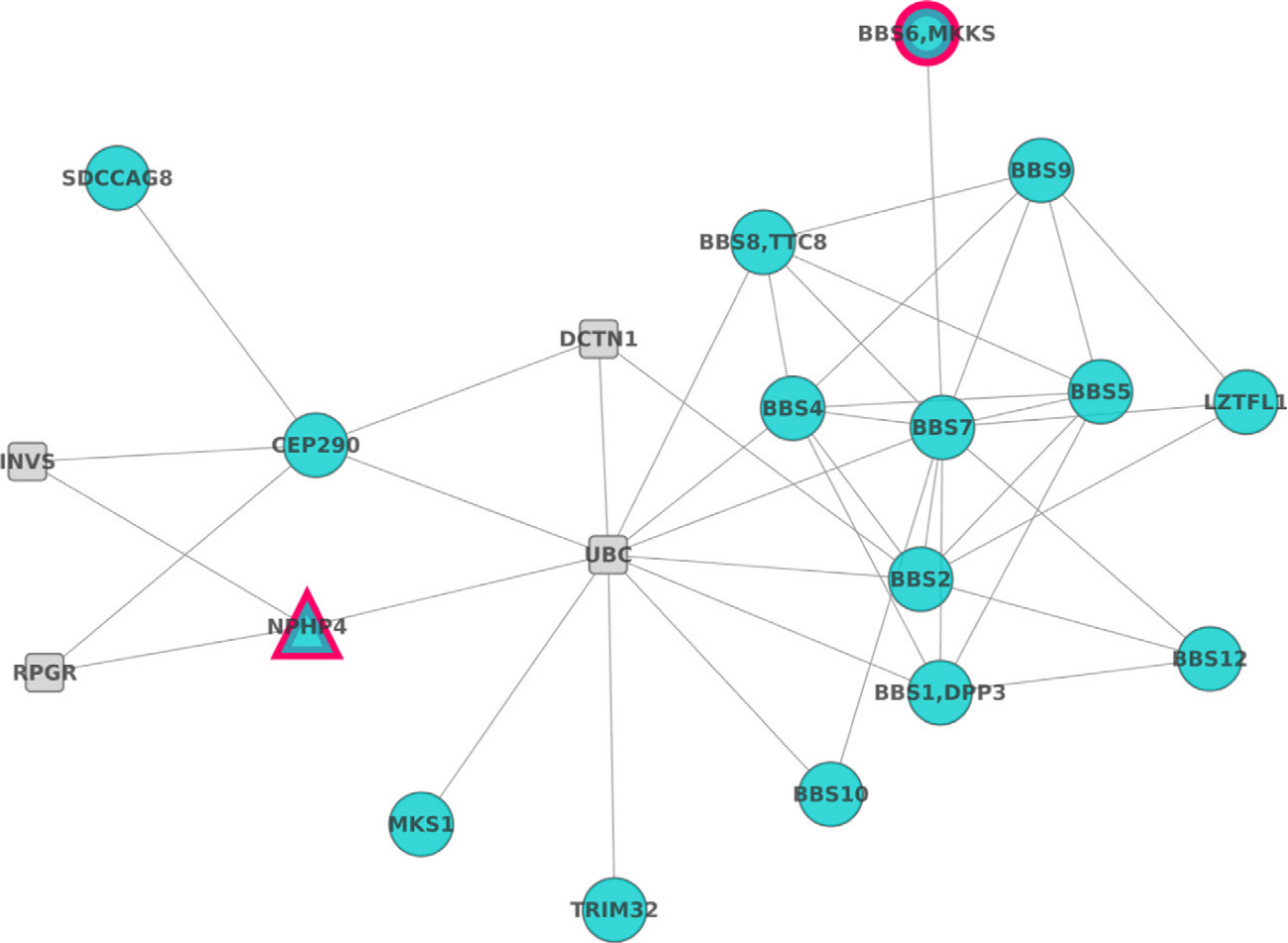
Significant protein–protein interaction network: Network analysis allows relating *NPHP4* to BBS proteins, supporting in this way its' possible role as modifier gene.

In order to explore the possible physical interactions among the BBS proteins and *NPHP4*, network enrichment analysis, using the program SNOW (Minguez et al. 2009), has been performed. Figure 4 shows the proteins significantly connected (significantly smallest number of components, *P*-value = 0.01) allowing the introduction of one extra connecting node among the proteins studied.

The network documents the dense network of physical protein–protein interactions that connect BBS proteins and also documents how *NPHP4* connects to several BBS proteins through different intermediates. Of special interest is *RPGR*, which connects *NPHP4* to *CEP290*. *RPGR*, the RP GTPase regulator, is also involved in RP X-Linked, cone-rod dystrophy X-linked, and macular dystrophy X-linked. Another interesting gene is *INVS*, Inversin, which encodes a protein that protein may function in renal tubular development and function. Such connections reinforce the possible role of *NPHP4* as modulator of the penetrance of the disease.

## Discussion

In this report, a Spanish BBS family with three affected siblings is described. The mode of inheritance and the main clinical features correspond to autosomal recessive BBS.

Exome analysis of four individuals (I:1, I:2, II:3, and II:4) led to the identification of two novel compound heterozygous mutations in the *MKKS* gene (c.269A>G; p.D90G and c.1186G>T; p.V396F) of affected individuals. These variants were absent in 200 control individuals and showed segregation with disease in the entire family. Mutation D90G lay within the predicted equatorial domain, the most conserved region among group I and II chaperonins. In the group II chaperonin family, this domain is responsible for ATP hydrolysis and the substitution might alter its structure. Mutation V396F lay within the predicted intermediate domain which connects the equatorial and the apical domains via flexible hinges (Stone et al. 2000). Bioinformatic analysis predicted pathogenic consequences for both missense mutations.

The molecular diagnosis is generally helpful to confirm a clinical diagnosis. Although many studies have dissected the clinical overlaps due to *MKKS* mutations, genotype–phenotype correlations are still not well understood. The clinical evaluation of the affected members of the family did not reveal hydrometrocolpos, suggesting that these two missense mutations in the *MKKS* gene did not cause genital malformations typical of MKKS phenotype. In this family, mutations in *MKKS* resulted in a spectrum of BBS phenotypes, ranging from BBS with severe renal involvement in patient II:4 to milder forms of BBS (patients II:2 and II:5). The expression of the phenotype and the disease progression can vary greatly from patient to patient, even among members of the same family. Whereas retinal dystrophy, digit anomaly, and learning disabilities were highly penetrant traits for all patients, kidney abnormalities and obesity showed incomplete penetrance in our family.

The index patient investigated here had previously undergone selected genotyping (APEX analysis, Asper http://www.asperbio.com/asper-ophthalmics), but this mutational screening approach failed to identify the underlying gene defect in this family. The implementation of a reliable diagnostic system able to detect novel disease-causing mutations, even in genes not previously associated with an assumed clinical diagnosis, is necessary. Exome sequencing has proven to be an important diagnostic tool for disorders that are characterized by significant genetic heterogeneity. In the case of BBS, besides the high number of genes involved, oligogenic inheritance is also well documented (Katsanis 2004) adding a layer of complexity to the genetic characterization of such patients.

One advantage of analyzing the whole exome simultaneously is the unique possibility to investigate the involvement of second-site mutations which may be modulating the expression of the BBS phenotype (Katsanis et al. 2001; Badano et al. 2003; Beales et al. 2003; Katsanis 2004; Hjortshoj et al. 2010). However, the use of unbiased methodologies often produces many candidates that must be filtered out with *in silico* prioritization techniques. Thus, network analysis captures the relationships of mutated genes with already known disease genes allowing a rational prioritization of candidate genes. The combination of NGS with network analysis approach conducted here allowed us to identify a novel variant – located on intron 8-9 of *NPHP4 –* which was predicted to enhance the use of a cryptic splice acceptor site. This would probably cause the introduction of a premature termination codon and the reduction in NPHP4 mRNA levels. Only the most severely affected patient (II:4) carries the variant (c.992 + 71G>T) in heterozygous state. In addition, the absence of the variant in control individuals seems to indicate that it is a pathogenic allele.

NPHP4 is a ciliary protein that is known to belong to a multifunctional complex and colocalizes with RPGRIP1, RPGR, and the serologically defined colon cancer antigen-8 (SDCCAG8), a protein thought to partake in the RPGRIP1 interactome and implicated also in retinal–renal ciliopathies (Roepman et al. 2005; Schaefer et al. 2011; Won et al. 2011; Patil et al. 2012). Mutations in *NPHP4* have been associated with related ciliopathies such as NPH (Mollet et al. 2002; Hoefele et al. 2004) and SLS (Otto et al. 2002; Schuermann et al. 2002).

Few splice site variants have been found to modulate the pathogenesis of BBS. One example is the allele C430T of *MGC1203* which enhances the use of a cryptic splice acceptor site, causing the introduction of a premature termination codon and the reduction in the steady-state *MGC1203* mRNA levels (Badano et al. 2006b). Although the MGC1203 mutations are probably insufficient to cause BBS, this gene may be involved in the pathogenesis of BBS by contributing hypomorphic mutations to an already sensitized genetic background. The authors conclude that suppression of *MGC1203* exerts an epistatic effect on the developmental BBS phenotype. It is tempting to speculate that *NPHP4* allele reported herein may also show a similar effect. We hypothesize that the *NPHP4* variant may be acting as a second-site modifier affecting the likelihood of renal degeneration in the context of the other *MKKS* mutations. This hypothesis is also supported by the known physical relationships revealed by network analysis. The MKKS protein forms a significant cluster of proteins with several BBS proteins, as shown in Figure 4. The severe BBS phenotype observed in patient II:4 may be due to an epistatic effect of *MKKS* and *NPHP4* mutations. This would provide an explanation for one of the distinct clinical manifestations observed in this family. Moreover, it is possible that other intrafamilial phenotypic variations may be due to alterations in additional loci as well as nonstrictly genetic factors not evaluated in this study. Therefore, our findings warrant further studies to evaluate the role of putative disease-modifying variants.

Identifying the presence of epistatic variants can be challenging, given that a large number of common and rare alleles – located in known and in novel genes – has been shown to influence the expressivity of the BBS phenotype. Our data demonstrate that NGS is a powerful approach to identify rare and high penetrant disease variants and to study the genetic contribution to phenotypic variability.

This genetic diagnostic tool can now be applied to large cohorts of Mendelian and oligogenic disorders and should rapidly provide the molecular diagnosis and the prevalence of associated genes.

In summary, we show how WES has clearly improved the molecular diagnosis of heterogeneous disorders such as BBS. In addition, our study highlights the usefulness of NGS approaches for the dissection of epistatic phenomena and provides promising findings to decipher the genetic basis of phenotypic variability.

## Supporting Information

Table S1 Variants identified in the index patient by exome sequencing in other related loci.
