# Supplementary material for: Deciphering intrafamilial phenotypic variability by exome sequencing in a Bardet–Biedl family
Source: Mol Genet Genomic Med. 2013 Dec 3;2(2):124–33. doi: 10.1002/mgg3.50 (PMC3960054; doi:10.1002/mgg3.50)
Supplement: Table S1 — Variants identified in the index patient by exome sequencing in other related loci. [file mgg30002-0124-sd1.doc]

**Supporting Table S1: Variants identified in the index patient by exome sequencing in other related loci.**

| **Gene** | **Genomic position (Hg 19)** | **RS ID/ Novel** | **Genotype** |
| --- | --- | --- | --- |
| *BBS2* | 16:56545175T>C | rs11373 | [M]+[=] |
| *BBS4* | 15:72987588G>T | rs4777527 | [M]+[M] |
| *BBS4* | 15:73002035G>A | rs8033604 | [M]+[M] |
| *BBS4* | 15:73007893G>A | rs730180 | [M]+[M] |
| *BBS4* | 15:73023937T>C | rs12914333 | [M]+[M] |
| *BBS4* | 15:73027478T>C | rs2277598 | [M]+[=] |
| *BBS4* | 15:73028375G>A | rs12324395 | [M]+[M] |
| *BBS5* | 2:170336024G>C | rs1879466 | [M]+[M] |
| *BBS5* | 2:170361415A>G | rs12692910 | [M]+[M] |
| *BBS5* | 2:170361426T>C | rs12692911 | [M]+[M] |
| *BBS6/MKKS* | 20:10386013C>A | rs1545 | [M]+[=] |
| *BBS6/MKKS* | 20:10386059G>A | rs1547 | [M]+[=] |
| *BBS6/MKKS* | 20:10388350C>A | Novel pathogenic | [M]+[=] |
| *BBS6/MKKS* | 20:10388506G>A | rs6133907 | [M]+[M] |
| *BBS6/MKKS* | 20:10389218T>C | rs6133909 | [M]+[=] |
| *BBS6/MKKS* | 20:10389480T>A | rs764266 | [M]+[=] |
| *BBS6/MKKS* | 20:10393162A>C | rs6133917 | [M]+[=] |
| *BBS6/MKKS* | 20:10393629G>A | rs17852625 | [M]+[=] |
| *BBS6/MKKS* | 20:10393894T>C | Novel pathogenic | [M]+[=] |
| *BBS6/MKKS* | 20:10394046G>A | rs16991547 | [M]+[=] |
| *BBS7* | 4:122749541C>T | rs1507994 | [M]+[=] |
| *BBS8/TTC8* | 14:89307320T>C | rs17700296 | [M]+[=] |
| *BBS9* | 7:33233240A>G | rs10270013 | [M]+[M] |
| *BBS9* | 7:33282577G>A | rs7793096 | [M]+[M] |
| *BBS9* | 7:33380598G>T | rs11981364 | [M]+[=] |
| *BBS9* | 7:33380673T>C | rs10486527 | [M]+[=] |
| *BBS9* | 7:33385852C>T | rs6974593 | [M]+[M] |
| *BBS9* | 7:33388618C>T | rs9785054 | [M]+[M] |
| *BBS9* | 7:33388829T>A | rs73101660 | [M]+[=] |
| *BBS11/TRIM32* | 9:119463189T>C | rs2281627 | [M]+[=] |
| *BBS13/MKS1* | 17:56290253C>T | rs115496661 | [M]+[=] |
| *BBS13/MKS1* | 17:56290253C>T | rs115496661 | [M]+[=] |
| *BBS14/CEP290* | 12:88453608C>T | rs2471512 | [M]+[=] |
| *BBS14/CEP291* | 12:88472996C>T | rs61941020 | [M]+[=] |
| *BBS14/CEP292* | 12:88496838C>G | rs2471532 | [M]+[=] |
| *BBS14/CEP293* | 12:88505078T>C | rs2468255 | [M]+[=] |
| *BBS16/SDCCAG8* | 1:243419429T>A | rs3904682 | [M]+[=] |
| *BBS16/SDCCAG9* | 1:243471192A>G | rs953492 | [M]+[=] |
| *BBS16/SDCCAG10* | 1:243481431A>G | rs2783972 | [M]+[=] |
| *BBS16/SDCCAG11* | 1:243487371A>G | rs2484638 | [M]+[M] |
| *BBS16/SDCCAG12* | 1:243493907A>T | rs2275155 | [M]+[=] |
| *BBS16/SDCCAG13* | 1:243579112G>A | rs10927011 | [M]+[=] |
| *BBS16/SDCCAG14* | 1:243651806C>T | rs12042298 | [M]+[=] |
| *INPP5* | 9:139323201A>G | rs1128877 | [M]+[=] |
| *INPP5* | 9:139323311T>C | rs8413 | [M]+[=] |
| *INPP5* | 9:139324680C>T | rs4451431 | [M]+[=] |
| *INPP5* | 9:139324740C>T | rs10870182 | [M]+[=] |
| *INPP5* | 9:139327034A>G | rs10870194 | [M]+[=] |
| *INPP5* | 9:139327439A>G | rs10781542 | [M]+[=] |
| *INPP5* | 9:139328722G>T | rs7851507 | [M]+[=] |
| *PKD1* | 16:2160494C>T | rs79884128 | [M]+[M] |
| *PKD2* | 4:88959381G>A | rs2725221 | [M]+[=] |
| *PKHD1* | 6:51483961T>C | rs9381994 | [M]+[=] |
| *PKHD1* | 6:51491884T>C | rs4715227 | [M]+[=] |
| *PKHD1* | 6:51492046T>G | rs9474033 | [M]+[=] |
| *PKHD1* | 6:51512707T>C | rs2580009 | [M]+[=] |
| *PKHD1* | 6:51524403G>A | rs34460237 | [M]+[=] |
| *PKHD1* | 6:51538501G>A | rs62461292 | [M]+[M] |
| *PKHD1* | 6:51586771G>A | rs9349593 | [M]+[M] |
| *PKHD1* | 6:51613177C>T | rs765525 | [M]+[M] |
| *PKHD1* | 6:51618599G>A | rs6914483 | [M]+[M] |
| *PKHD1* | 6:51619890G>A | rs9395712 | [M]+[M] |
| *PKHD1* | 6:51628525T>C | rs9382015 | [M]+[M] |
| *PKHD1* | 6:51640751C>G | rs3920621 | [M]+[M] |
| *PKHD1* | 6:51649079A>G | rs4715232 | [M]+[M] |
| *PKHD1* | 6:51656236G>T | rs7765455 | [M]+[M] |
| *PKHD1* | 6:51682966C>T | rs7748507 | [M]+[M] |
| *PKHD1* | 6:51712492A>T | rs9370067 | [M]+[=] |
| *PKHD1* | 6:51720838T>C | rs9349603 | [M]+[=] |
| *PKHD1* | 6:51720872A>G | rs7452724 | [M]+[=] |
| *PKHD1* | 6:51732598G>A | rs9382043 | [M]+[=] |
| *PKHD1* | 6:51732628G>A | rs9382044 | [M]+[=] |
| *PKHD1* | 6:51732807C>T | rs12210295 | [M]+[=] |
| *PKHD1* | 6:51735261G>T | rs982874 | [M]+[=] |
| *PKHD1* | 6:51798753G>A | rs1266888 | [M]+[=] |
| *PKHD1* | 6:51798760T>A | rs1884953 | [M]+[=] |
| *PKHD1* | 6:51799166T>C | rs1266889 | [M]+[=] |
| *PKHD1* | 6:51875250A>C | rs2435322 | [M]+[M] |
| *PKHD1* | 6:51917968T>G | rs4715271 | [M]+[=] |
| *PKHD1* | 6:51923409A>T | rs4715272 | [M]+[=] |
| *PKHD1* | 6:51924774A>G | rs1896976 | [M]+[M] |
| *PKHD1* | 6:51935061T>G | rs4711996 | [M]+[M] |
| *PKHD1* | 6:51938242A>G | rs9474140 | [M]+[=] |
| *PKHD1* | 6:51947237G>A | rs9474143 | [M]+[=] |
| *PKHD1* | 6:51947257G>A | rs6901799 | [M]+[=] |
| *NPHP1* | 2:110882699A>G | rs2048243 | [M]+[M] |
| *NPHP1* | 2:110886679A>G | Novel intronic | [M]+[=] |
| *NPHP1* | 2:110886683C>T | rs111532194 | [M]+[=] |
| *NPHP1* | 2:110889108C>T | rs11240790 | [M]+[M] |
| *NPHP1* | 2:110902262G>A | rs3752863 | [M]+[M] |
| *NPHP1* | 2:110905664G>A | rs1509417 | [M]+[M] |
| *NPHP1* | 2:110906415T>C | rs7599788 | [M]+[M] |
| *NPHP1* | 2:110919321C>T | rs2271244 | [M]+[M] |
| *NPHP1* | 2:110922703C>T | rs11675767 | [M]+[M] |
| *NPHP1* | 2:110942150G>A | rs881302 | [M]+[M] |
| *NPHP1* | 2:110942496G>A | rs906815 | [M]+[M] |
| *NPHP1* | 2:110942656A>G | rs2018711 | [M]+[M] |
| *NPHP1* | 2:110942694T>C | rs1810097 | [M]+[M] |
| *NPHP1* | 2:110942816G>C | rs1810096 | [M]+[M] |
| *NPHP1* | 2:110959898C>G | rs11240794 | [M]+[M] |
| *INVS* | 9:102869846G>A | rs10988973 | [M]+[M] |
| *INVS* | 9:103015184T>C | rs2245216 | [M]+[M] |
| *INVS* | 9:103054941G>A | rs76868679 | [M]+[=] |
| *INVS* | 9:103054951T>C | rs2787374 | [M]+[M] |
| *NPHP3* | 3:132435532C>T | rs67092166 | [M]+[=] |
| *NPHP3* | 3:132435823C>T | rs11718115 | [M]+[=] |
| *NPHP3* | 3:132436967A>G | rs66564593 | [M]+[=] |
| *NPHP4* | 1:5923788T>G | rs11121648 | [M]+[=] |
| *NPHP4* | 1:5934490A>G | rs868163 | [M]+[M] |
| *NPHP4* | 1:5935162A>T | rs1287637 | [M]+[M] |
| *NPHP4* | 1:5935222T>C | rs963030 | [M]+[=] |
| *NPHP4* | 1:5937091T>C | rs1622955 | [M]+[M] |
| *NPHP4* | 1:5937391C>T | rs3747989 | [M]+[M] |
| *NPHP4* | 1:5939544T>G | rs11121915 | [M]+[M] |
| *NPHP4* | 1:5948677G>A | rs2282281 | [M]+[M] |
| *NPHP4* | 1:5964991T>C | rs3737234 | [M]+[M] |
| *NPHP4* | 1:5967064G>T | rs9628990 | [M]+[M] |
| *NPHP4* | 1:6007446C>T | rs551207 | [M]+[M] |
| *NPHP4* | 1:6008059C>A | Novel splicing | [M]+[=] |
| *NPHP4* | 1:6027167C>A | rs875574 | [M]+[M] |
| *NPHP4* | 1:6027252A>G | rs875573 | [M]+[M] |
| *NPHP4* | 1:6038583G>C | rs4908639 | [M]+[M] |
| *IQCB1* | 3:121500699C>T | rs17849995 | [M]+[=] |
| *IQCB1* | 3:121526099A>G | rs4582090 | [M]+[=] |
| *IQCB1* | 3:121526204G>A | rs4543051 | [M]+[=] |
| *GLIS2* | 16:4386814T>C | rs669561 | [M]+[=] |
| *GLIS2* | 16:4388139G>A | rs653539 | [M]+[=] |
| *RPGRIP1L* | 16:53634295C>T | rs1946155 | [M]+[=] |
| *RPGRIP1L* | 16:53635933A>T | rs4784319 | [M]+[=] |
| *RPGRIP1L* | 16:53672355C>T | rs7203525 | [M]+[=] |
| *RPGRIP1L* | 16:53699003G>A | rs4133017 | [M]+[=] |
| *NEK1* | 4:170322819G>A | rs200710438 | [M]+[=] |
| *NEK1* | 4:170354836C>T | rs7680152 | [M]+[=] |
| *NEK1* | 4:170354873T>C | rs13139925 | [M]+[=] |
| *NEK1* | 4:170398454T>C | rs34099167 | [M]+[=] |
| *NEK1* | 4:170428331G>T | rs6855803 | [M]+[=] |
| *NEK1* | 4:170428843C>A | rs17054977 | [M]+[=] |
| *NEK1* | 4:170429359C>T | rs66509122 | [M]+[=] |
| *NEK1* | 4:170482883A>G | rs56064008 | [M]+[=] |
| *NEK1* | 4:170501850A>T | rs7665092 | [M]+[=] |
| *NEK1* | 4:170501954T>C | rs7670271 | [M]+[=] |
| *NEK1* | 4:170506703A>G | rs55679731 | [M]+[=] |
| *NEK1* | 4:170532808A>G | rs28439336 | [M]+[M] |
| *TMEM67/MKS3* | 8:94768124C>G | rs74712633 | [M]+[=] |
| *TMEM67/MKS3* | 8:94794620C>T | rs3097427 | [M]+[=] |
| *TMEM67/MKS3* | 8:94808165A>G | rs3134031 | [M]+[=] |
